# Highly accelerated vessel‐selective arterial spin labeling angiography using sparsity and smoothness constraints

**DOI:** 10.1002/mrm.27979

**Published:** 2019-09-19

**Authors:** S. Sophie Schauman, Mark Chiew, Thomas W. Okell

**Affiliations:** ^1^ Wellcome Centre for Integrative Neuroimaging FMRIB, Nuffield Department of Clinical Neurosciences University of Oxford Oxford United Kingdom

**Keywords:** arterial spin labelling, compressed sensing, dynamic angiography, radial golden angle, vessel‐encoded ASL

## Abstract

**Purpose:**

To demonstrate that vessel selectivity in dynamic arterial spin labeling angiography can be achieved without any scan‐time penalty or noticeable loss of image quality compared with conventional arterial spin labeling angiography.

**Methods:**

Simulations on a numerical phantom were used to assess whether the increased sparsity of vessel‐encoded angiograms compared with non‐vessel‐encoded angiograms alone can improve reconstruction results in a compressed‐sensing framework. Further simulations were performed to study whether the difference in relative sparsity between nonselective and vessel‐selective dynamic angiograms was sufficient to achieve similar image quality at matched scan times in the presence of noise. Finally, data were acquired from 5 healthy volunteers to validate the technique in vivo. All data, both simulated and in vivo, were sampled in 2D using a golden‐angle radial trajectory and reconstructed by enforcing image domain sparsity and temporal smoothness on the angiograms in a parallel imaging and compressed‐sensing framework.

**Results:**

Relative sparsity was established as a primary factor governing the reconstruction fidelity. Using the proposed reconstruction scheme, differences between vessel‐selective and nonselective angiography were negligible compared with the dominant factor of total scan time in both simulations and in vivo experiments at acceleration factors up to R = 34. The reconstruction quality was not heavily dependent on hand‐tuning the parameters of the reconstruction.

**Conclusion:**

The increase in relative sparsity of vessel‐selective angiograms compared with nonselective angiograms can be leveraged to achieve higher acceleration without loss of image quality, resulting in the acquisition of vessel‐selective information at no scan‐time cost.

## 1 INTRODUCTION

Angiographic methods are used to detect vascular abnormalities such as aneurysms, atherosclerosis, and arteriovenous malformations. However, many of the commonly used techniques for imaging the cerebral vasculature have associated risks. Digital subtraction angiography involves ionizing radiation and risk of complications.^1^ In contrast‐enhanced magnetic resonance angiography (MRA), there are also concerns associated with gadolinium‐based contrast agents, which are unsuitable for patients with renal dysfunction^2^ and have been shown to be retained in the brain.^3^

Arterial spin labeling (ASL) can be used for non‐contrast‐enhanced MRA. Compared with other non‐contrast‐enhanced MRA methods, ASL is a more versatile and flexible technique. For example, ASL can provide dynamic information about blood flow. Because of the complete removal of background tissue signal by subtraction of “label” and “control” images, smaller vessels can be resolved using ASL compared with time‐of‐flight angiography at the same resolution.^4^ Arterial spin labeling can also provide vessel‐specific angiograms, whereas other MR methods generally provide only nonspecific angiograms. Vessel specificity and dynamic information can be crucial in planning for surgical interventions and predicting clinical outcomes, such as in assessing collateral flow in stroke.^5^, ^6^

One way of achieving vessel selectivity with ASL is using vessel‐encoded ASL (VE‐ASL),^7^ which is generally implemented as a variant of pseudo‐continuous ASL^8^ and is the focus of this study. Similar encoding methods have also been proposed using pulsed ASL, and although pulsed ASL benefits from shorter labeling durations, vessel geometries often result in unintended tagging of multiple arteries.^9^ Compared with vessel‐selective ASL methods that label only 1 vessel at a time,^10^, ^11^ VE‐ASL is more SNR‐efficient, as all acquired data inform all vessel‐selective images. The SNR of a decoded VE image is the same as for a non‐vessel‐encoded (non‐VE) image acquired at the same scan time for fully sampled data.^7^ The main drawback of VE‐ASL is longer acquisition times compared with non‐VE‐ASL. This is because *N* + 1 images are required to separate blood coming from *N* arteries compared with only a tag and a control image for non‐VE‐ASL. To achieve matched scan time with non‐VE acquisitions, the VE images have to be acquired with higher undersampling.

However, we hypothesized that VE‐ASL can be highly accelerated using nonlinear reconstruction methods. Two main reasons why dynamic VE‐ASL angiograms might be particularly well‐suited to undersampled reconstruction are related to the intrinsic properties of angiographic data. First, angiograms are spatially sparse. This can be exploited in a compressed‐sensing^12^, ^13^ acquisition and reconstruction framework. Compared with non‐VE‐ASL angiograms, VE‐ASL images have higher relative sparsity, because approximately the same number of nonzero voxels are distributed across multiple vessel‐selective images. Because relative sparsity, along with image dimensionality and SNR, contribute to the performance of a compressed‐sensing reconstruction,^14^ we hypothesized that VE‐ASL angiograms could perform better than non‐VE angiograms in a sparsity‐constrained reconstruction. Second, at sufficiently high temporal resolution, the signal varies smoothly in time^15^ as the bolus of labeled blood passes through the arterial tree. This temporal smoothness can be exploited to further regularize the underdetermined image reconstruction problem. Although exploiting redundancy in the temporal domain is possible in the dynamic acquisitions provided by both VE‐ASL and non‐VE ASL, nondynamic methods like time‐of‐flight angiography cannot benefit from this extra dimension of information.

In this study, we present an accelerated acquisition and reconstruction method for dynamic VE‐ASL angiography based on the enhanced spatial sparsity of vessel‐specific angiograms and the smoothness of their temporal evolution. We demonstrate that the proposed method produces VE‐ASL images of comparable quality to non‐VE‐ASL at matched scan duration at acceleration factors varying from R = 2 to R = 34, providing vessel‐specific information at no additional cost.

## 2 METHODS

### 2.1 Modeling the imaging system

**Figure 1:**
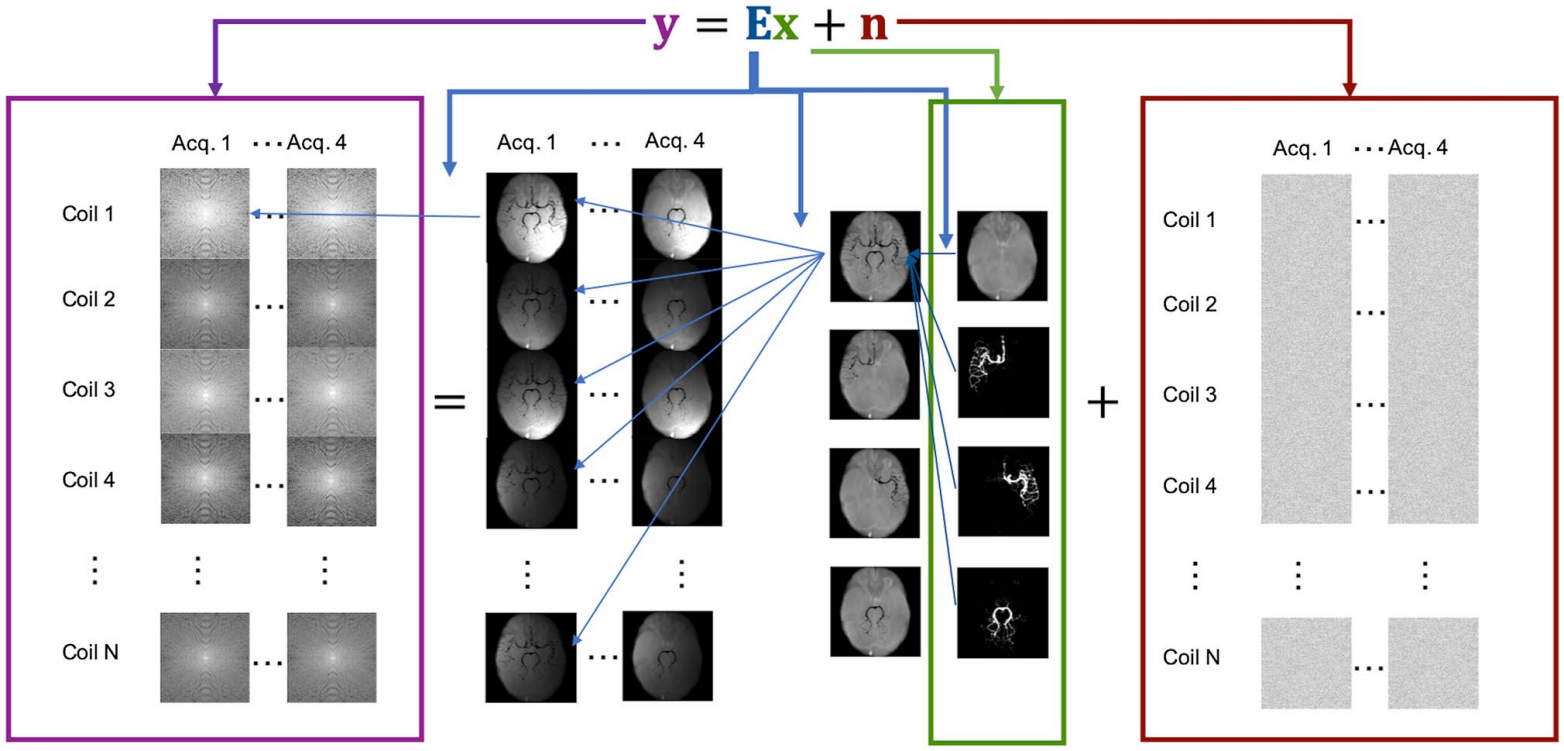
Model of imaging system used in simulations and reconstructions. ***y*** (purple box) represents the raw data that are a combination of noise (***n***, red box) and the object (***x***, green box) after being transformed by the imaging system (***E***, blue arrows), which consists of 3 parts (right to left): vessel‐encoding (VE), application of coil sensitivities, and an undersampled Fourier transform from physical space to k‐space

The imaging system (Figure 1) was modeled as a linear equation as follows:

(1) y=Ex+n

where ***y*** is a vector containing the complex signal measured by all of the receive coils, with each entry representing 1 point in k‐space in 1 coil. Noise, ***n***, is a vector of complex white noise. The imaged object, ***x***, is a vector containing the complex magnetization of blood from each vessel component as well as the static tissue for every position in physical space. Its length is therefore the number of voxels by the number of time points by the number of vessel components (i.e., a 3‐vessel VE image would have 4 components [3 vessels and static tissue] and a non‐VE image only 2 components [vessels and static tissue], thus making ***x*** twice as long in the VE case). In this work, a 3‐vessel VE image was used, separating blood originating from 3 arteries: the right and left internal carotid arteries (RICA and LICA, respectively) and the basilar artery (BA). ***E*** is the linear encoding operator that models (1) the linear combination of signal from blood and static tissue depending on the applied VE scheme, (2) the multiplication of spatial sensitivity profiles for each of the receive coils in the system, and (3) the k‐space sampling transform.

Simulations of the imaging system and subsequent reconstruction of both simulated and in vivo data were performed using MATLAB (Release 2017a; MathWorks, Natick, MA). The applied VE scheme of ***E*** was implemented directly as a matrix multiplication of either a 2 × 2 Hadamard matrix (1 image with labeled blood and 1 control image) for the non‐VE case or a 4 × 4 Hadamard matrix for the VE case, as follows:

(2) $$\text{nonVE}:\left[\begin{array}{cc} -1 & 1\\ 1 & 1 \end{array}\right]\left[\begin{array}{c} \text{RICA}+\text{LICA}+\text{BA}\\ S \end{array}\right]$$

(3) $$\text{VE}:\left[\begin{array}{cccc} -1 & -1 & -1 & 1\\ -1 & 1 & 1 & 1\\ 1 & -1 & 1 & 1\\ 1 & 1 & -1 & 1 \end{array}\right]\left[\begin{array}{c} \text{RICA}\\ \text{LICA}\\ \text{BA}\\ S \end{array}\right]$$

where RICA, LICA, and BA represent the signal from the blood coming from the respective arteries, and S represents the static tissue signal.

The coil sensitivities and their conjugate transposes were applied as point‐wise multiplication on the image and weighted combination of coils for the forward and adjoint transform, respectively. The transforms between nonuniform k‐space samples and image space were implemented using the nonuniform fast Fourier transform^16^ in the Michigan Image Reconstruction Toolbox.^17^ In our method, we used a golden‐angle radial trajectory.^18^

### 2.2 Simulations

Two data sets were used as ground truths for numerical simulations. One was a numerical phantom and the other a fully sampled dynamic VE‐ASL angiogram from a previous study.^4^ The numerical phantom consisted of a single frame of a hand‐drawn “vessel‐like” image on a 96 × 96 pixel grid. It consisted of 3 vessel components and a static tissue component. One vessel component occupied primarily the left FOV, a mirror image of it occupied primarily the right FOV, and the third component occupied the lower space between the two. In 9 pixels, 2 vessel components overlapped. The vessel structure had voxel intensities ranging from 0.54 to 1.00, and the static tissue component was a circle covering all of the “vessels,” with uniform intensity of 100.00. It was used to confirm that the initial hypothesis (that increased relative sparsity improves the reconstruction) holds in a simplified system. The non‐VE data had 14% nonzero values in the vessel image, whereas the VE data contained only 5% nonzero values in the 3 vessel images combined. These levels of sparsity are realistic for angiographic imaging at or just above the circle of Willis, as confirmed by the in vivo data acquisition presented in section 2.3 that had sparsity levels of 5.3% ± 0.7% for VE and 15.2% ± 2.2% for non‐VE. The noise was set to zero, and only 1 receive coil with uniform spatial sensitivity was modeled. The images were transformed into k‐space data using the forward model described in section 2.1 and reconstructed with 100%, 50%, 25%, 12.5%, 6.25%, and 3.125% of the number of samples needed to reach the Nyquist limit.

A real, high SNR dynamic angiogram was used to mimic the in vivo system as closely as possibly but with a well‐defined ground truth and controlled noise conditions. Coil sensitivities previously measured using a phantom in a 32‐channel head coil were included to generate multichannel ground truth data in the simulated acquisition. The coil sensitivity profiles used in reconstruction were, however, estimated from the undersampled data directly, as explained further in section 2.4. Complex Gaussian noise was added in k‐space, and SNR was defined as

(4) $$SNR_{k}=\frac{\text{rms}\left(I\right)}{\sigma_{\text{noise}}},$$

where rms(*I*) represents the RMS intensity of the noiseless k‐space measurements, and *σ* is the SD of the added noise signal. Three noise conditions were simulated: (1) no noise, (2) moderate noise (*SNR_k_* = 185.8), and (3) high noise (*SNR_k_* = 92.9). These simulated data sets were subsequently undersampled and reconstructed in the same manner as the in vivo data (see section 2.5). The high noise condition produced image SNR comparable to the in vivo image SNR (Supporting Information Figure S1) when reconstructed with a linear least‐squares reconstruction on fully sampled data.

### 2.3 In vivo acquisition

Six healthy volunteers (all male, age range: 25 to 34, mean age: 29) were scanned on a 3T Magnetom Verio (Siemens Healthineers, Erlangen, Germany) MRI scanner using a 32‐channel head coil, with a dynamic 2D golden‐angle radial VE‐ASL and non‐VE‐ASL sequence, similar to the implementation described in Okell.^19^ Data from 5 subjects were used for the main comparison of VE and non‐VE at matched scan times, and 1 subject was used to study the generalizability of the method at higher spatial resolution. All in vivo data were acquired under a technical development protocol approved by the local ethics committee.

Labeling was performed with pseudo‐continuous ASL using transverse gradients (G_xy_) of alternating polarity applied between the RF pulses to modulate the inversion efficiency across the labeling plane.^7^ The labeling plane was set just below the confluence of left and right vertebral arteries. For this study, the vertebral arteries were treated as a single artery to allow a 4‐cycle Hadamard encoding scheme to be performed. More distal labeling in which the vertebral arteries merge to form the BA was not performed, to ensure artifacts associated with the labeling plane did not overlap with the imaging region.

**Figure 2:**
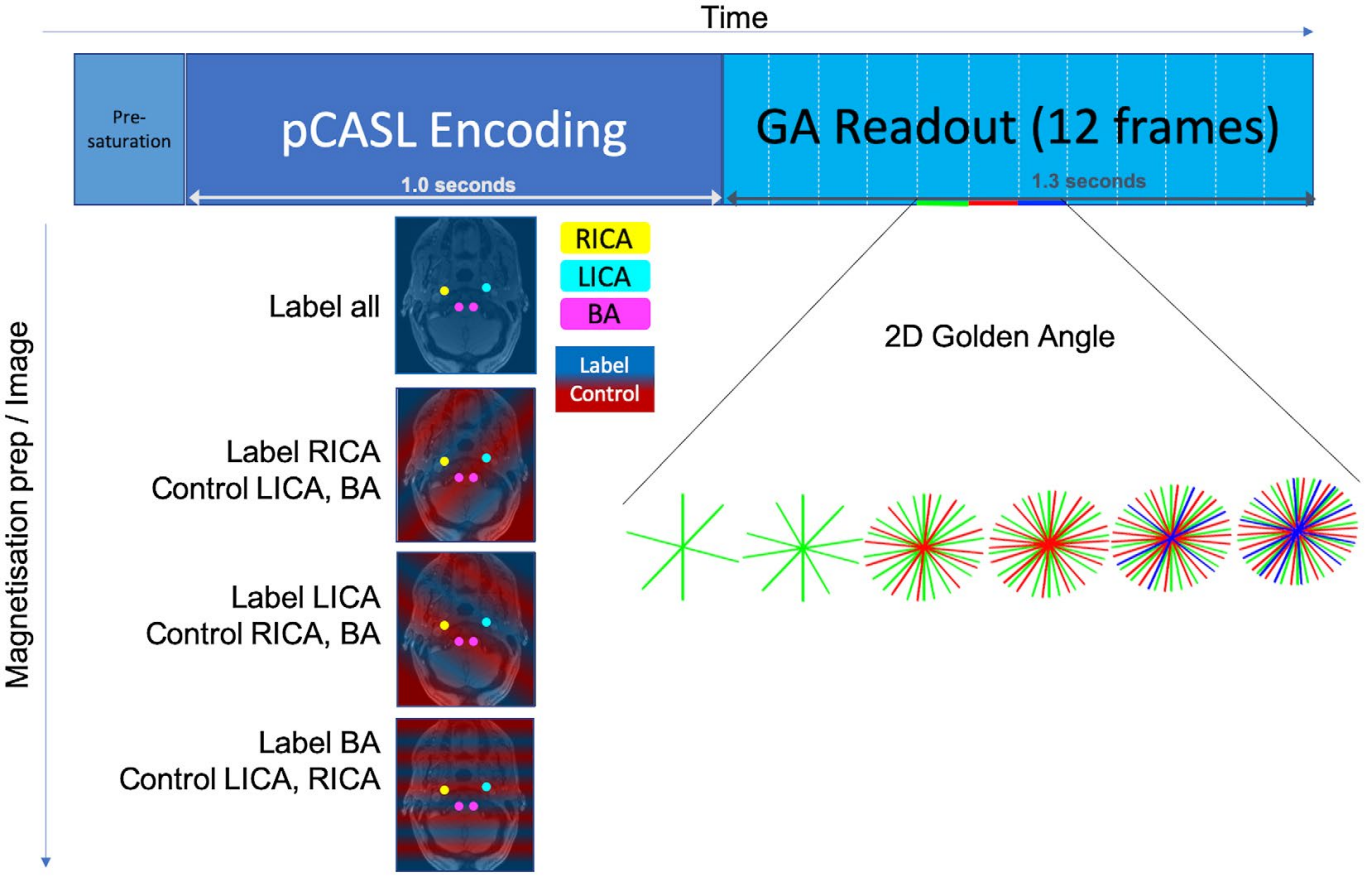
The imaging sequence consisted of a presaturation module for background suppression, pseudo‐continuous ASL (pCASL) labeling, and a spoiled gradient‐echo readout in a radial golden‐angle (GA) trajectory. The continuous readout was separated into frames in reconstruction. Four different magnetization preparation modules were used to encode the left and right internal carotid arteries (RICA, yellow; LICA, cyan) and the 2 vertebral arteries, which were close together and therefore encoded as a single vessel, referred to as the basilar artery (BA, magenta). This color scheme will be used in all subsequent figures. Transverse gradients within the pseudo‐continuous ASL pulse train generate spatial modulations to create tag (blue shading) and control (red shading) regions across the labeling plane

A spoiled gradient‐echo readout was initiated (TR = 11.73 ms, TE = 5.95 ms, flip angle = 7º) immediately after the pseudo‐continuous ASL labeling pulse train (labeling duration: 1000 ms) (Figure 2). Each preparation was preceded by a presaturation module for background suppression, and 108 radial spokes were read out during the 1266.8‐ms‐long imaging period. These 108 spokes were split into 12 phases of 9 spokes in the reconstruction. The same set of 108 spokes were read out for each encoding before moving on to the next set of 108 spokes that were ordered, such that each phase of 9 spokes carried on the golden‐angle ordering from the previous shot that had finished, as described in Okell.^19^ The images for the 5 subjects used to compare VE against non‐VE at matched scan time were reconstructed with 1.1 × 1.1 × 50.0 mm spatial resolution (matrix size 192 × 192) and 105.57‐ms temporal resolution. The high‐resolution data set was acquired with 0.68‐mm isotropic in‐plane resolution and a matrix size of 320 × 320. A total of 34 ASL preparations were needed for each encoding to fully sample the 1.1‐mm^2^ resolution images, and 56 for the 0.68‐mm^2^ resolution images. The ASL preparation module was repeated every 2400 ms, so for 34 shots and 4 encodings (VE) the total scan time to reach the Nyquist limit was 5 minutes 26 seconds, and for 2 encodings (non‐VE) the total scan time was 2 minutes 43 seconds. For the high‐resolution VE data (56 shots, 4 encodings), the total scan time for R = 1 was 8 minutes 58 seconds.

The acceleration factor, R, was defined in relation to the fully Nyquist‐sampled acquisition time. An oversampled 1.1‐mm^2^‐resolution data set (acquisition time 10 minutes 53 seconds) for both the non‐VE (R = 0.25) and VE (R = 0.5) cases were acquired to be used as ground truth. Then, independently acquired test data sets for both VE and non‐VE (total acquisition time 5 minutes 26 seconds each) were used to assess the reconstruction method. Before reconstructing, the test data were split into multiple subsets by grouping sequentially acquired spokes, such that the number of spokes in a group corresponded to a specific acceleration factor. For example, subset 1 at R = 2 would include the first 153 spokes in each frame (306 needed for R = 1), and subset 2 would include the following 153 spokes. The images were reconstructed at 3 different levels of acceleration with matched scan time between non‐VE and VE: (1) high undersampling: R = 34 for VE (maximum acceleration, as this used only 1 ASL preparation for each encoding) and R = 17 for non‐VE, scan time 10 seconds; (2) medium undersampling: R = 8.5 for VE and 4.25 for non‐VE, scan time 38 seconds; and (3) low undersampling: R = 2 for VE and R = 1 (no undersampling) for non‐VE, scan time 2 minutes 43 seconds.

### 2.4 Preprocessing

Because decoding of the signals was handled with complex data, they were sensitive to phase errors. Therefore, for the in vivo data, phase correction was applied to account for B_0_ drift during the scan by minimizing the phase difference between the same spokes (***k***_*n*_) in different encodings (*n* = {0,1,2,3}) with a scalar phase correction factor (*e* ^*iθn*^), as follows:

(5) $$\min_{\theta_{n}}{\left|k_{0}-k_{n}e^{i\theta_{n}}\right|}_{2}^{2}$$

To minimize, *θ*_*n*_ is chosen to be arg(***k*** *_0_^H^* ***k*** *_n_)*.

Coil‐sensitivity calibration images were reconstructed by combining k‐space data across temporal frames to give 1 fully sampled or near fully sampled image. This was then used to estimate the relative coil‐sensitivity profiles for every point in space using the adaptive combine method.^20^ These estimated coil‐sensitivity profiles were used for generating the encoding operator, ***E***, as defined in Equation 1, for each data set. To improve speed and reduce the memory burden of the reconstruction, the 32 coil channels were compressed to 8 channels using singular value decomposition.^21^

### 2.5 Reconstruction

Both the simulated and in vivo data were reconstructed using the same method. Image reconstruction was achieved by the optimization of a nonlinear cost function, as follows:

(6) $$c\left(x\right)=\frac{1}{2}{\left|\mathrm{Ex}-y\right|}_{2}^{2}+\lambda_{1}{\left|x\right|}_{1}+\frac{1}{2}\lambda_{2}{\left|\nabla_{t}x\right|}_{2}^{2}.$$

In the cost function, ***x*** is the unknown image (the vessel components and the static tissue at all time points concatenated), ***E*** is the image‐acquisition operator, ***∇***_*t*_ is the finite‐difference operator in the temporal domain, and ***y*** is the vector containing the raw k‐space data as defined in Equation 1. Here, the first term imposes data consistency of the reconstruction, the second term imposes sparsity, and the third term enforces temporal smoothness. The values of *λ*_1_ and *λ*_2_ weigh the importance of the regularizing terms against data consistency. This cost function was minimized using the fast iterative shrinkage thresholding algorithm^22^ using a Toeplitz embedding formulation to replace nonuniform fast Fourier transforms with fast Fourier transforms for reduced computation time.^23^

The regularization factors in Equation 5 were determined experimentally by a grid search across a range of potential values. The (*λ*_1_, *λ*_2_) search space was chosen to be wide enough to ensure it fully characterized the target optima. The value of *λ*_1_ was varied from 0 to 10^−5^ in steps of 10^−6^ for all acceleration factors both in vivo and for the simulated data. For the high undersampling (R = 17 and 34), *λ*_2_ was varied from 0 to 6 in vivo, and 0 to 2 in simulations, in steps of 0.2. For the medium undersampling (R = 4.25 and 8.5), *λ*_2_ was varied 0 to 10 in steps of 1 for both the in vivo and simulation case. For the low undersampling (R = 1 and 2), it was varied in steps of 2 from 0 to 20.

For the simulations, the combination of *λ*_1_ and *λ*_2_ with the highest correlation with the ground truth (as explained in section 2.6) for each acceleration factor and noise level was used. In vivo, the average performance across all subjects and 8 subsets of data (except for the vessel‐encoded R = 2 and non‐vessel‐encoded R = 1 case, in which only 2 subsets of data were acquired) were calculated and the regularization factors that produced the highest correlation score on average were chosen and used for further analysis. Subject‐specific optimal ranges of regularization factors, which resulted in less than 1% quality reduction from the subject‐specific optimum, were also calculated. This was done to confirm that the overall optimal combination of regularizing factors was reasonable for all subjects. The overlap of these subject‐specific optimal regularization ranges was also inspected to see how subject‐dependent the optimal regularization factors were.

### 2.6 Analysis

All reconstructions were compared against the ground‐truth images. For the simulations, the input image was used directly for comparison. For the in vivo data, the oversampled acquisition was reconstructed with minimal regularization applied for denoising (*λ*_1_ = 10^−6^, *λ*_2_ = 0) and used as ground truth.

For quantitative assessment of image quality, non‐overlapping vessel‐specific masks (Supporting information Figure S2) were applied to both the reconstruction and the ground truth, as it was important that the quantitative assessment focused on the relevant pixels in the sparse images to avoid bias due to artifacts (such as from eye motion). The masks were then applied to each frame of each vessel component (or the total vessel component for non‐VE). The Pearson correlation coefficient (*r*) between the ground truth and reconstructed pixel values across all time points within the masks were then calculated.

When comparing non‐VE against VE, the correlation coefficients, *r*, for each vessel mask were Fisher‐transformed to a *z*‐score to make the distributions of correlation coefficients more Gaussian. This then allowed Student *t* tests to be performed to determine statistical significance at a 98.3% confidence interval (95% with added Bonferroni correction for multiple comparisons of the 3 vessel components).

## 3 RESULTS

### 3.1 Effect of increased relative sparsity in numerical phantom

**Figure 3:**
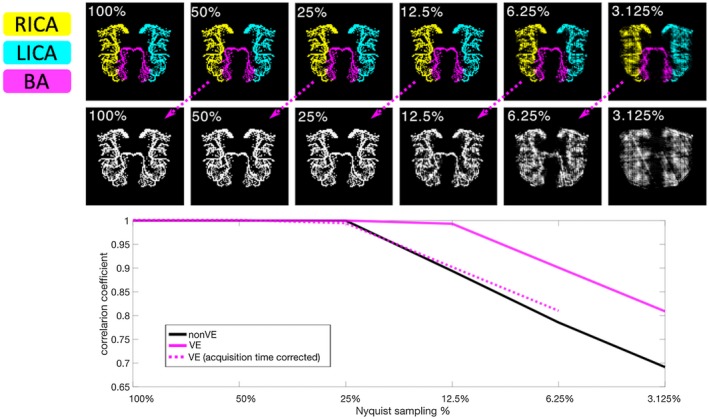
Numerical phantom simulations. Below 25% Nyquist sampling, the quality of the non‐vessel‐encoded (non‐VE) reconstruction decreases rapidly. For VE, this only occurs at twice the undersampling factor. The dashed line shows a shifted copy of the VE line, to illustrate the reconstruction quality of a 3‐vessel VE angiogram at the same acquisition time as the non‐VE. The corresponding time‐matched images (top row) are linked via dashed arrows. Reconstruction quality is quantified using the correlation coefficient between voxels in the reconstructed image and the ground truth (100% sampling). A version with the inverted grayscale contrast for direct comparison is available in Supporting Information Figure S3

In the numerical phantom, where the only difference between non‐VE and VE was the ratio of nonzero to zero voxels, the VE data were reconstructed more robustly at higher undersampling factors than non‐VE (Figure 3). The VE and non‐VE data could be reconstructed essentially perfectly at low undersampling factors, but for less than 25% of Nyquist sampling, the reconstruction of the sparser VE simulation outperformed the non‐VE version, achieving approximately matched performance for twice the undersampling factor, negating the factor of 2 time penalty that would be needed to perform 3‐vessel VE instead of non‐VE angiography. Qualitatively, increased blurring and streaking artifacts were observed in the non‐VE case.

### 3.2 Simulations on real data

**Figure 4:**
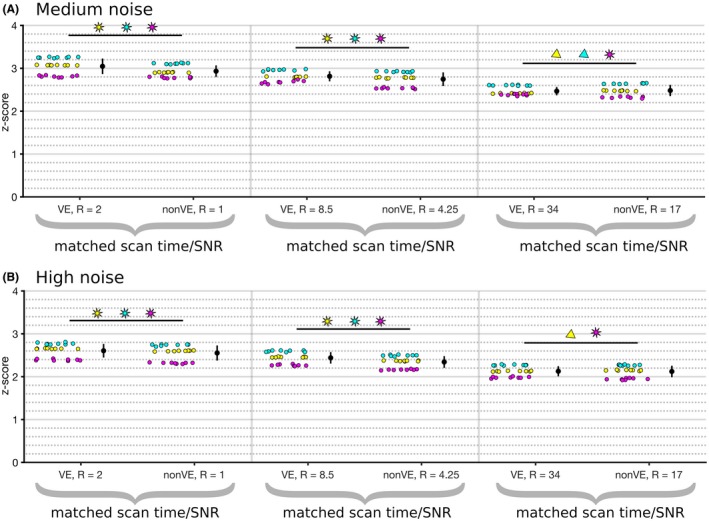
Reconstruction quality in simulations with medium noise (A) and high noise (B). Each scatter point represents the Fisher‐transformed correlation coefficient calculated in a mask (RICA, yellow; LICA, cyan; BA, magenta) for 1 reconstruction. Statistical significance between the time‐matched non‐VE and VE groups is represented by a star if VE had a higher correlation coefficient and a triangle if non‐VE did

Similar results to the numerical phantom were observed in the real data simulations (Figure 4). With no added noise, the VE and non‐VE reconstruction quality was high (*r* > 0.99) at all acceleration factors, and no difference was found between VE and non‐VE. With added noise and simulated matched scan times (equal SNR, but twice the undersampling for VE), VE was reconstructed marginally, but significantly, better (*P* < .01) than non‐VE for low and medium acceleration factors in both medium‐noise and high‐noise conditions. At high acceleration factors, the results varied more and VE performed better than non‐VE in some vessels but worse in others, and for some there was no statistically significant difference.

### 3.3 In vivo optimal regularization factors

The optimal regularization factors for the in vivo reconstructions did not vary considerably among different subjects, and their optimal ranges (within 1% of the optimum) had considerable overlap at all acceleration factors, and the group optimum was within the subject‐specific optimal ranges for both VE and non‐VE (Supporting Information Figure S4).

**Figure 5:**
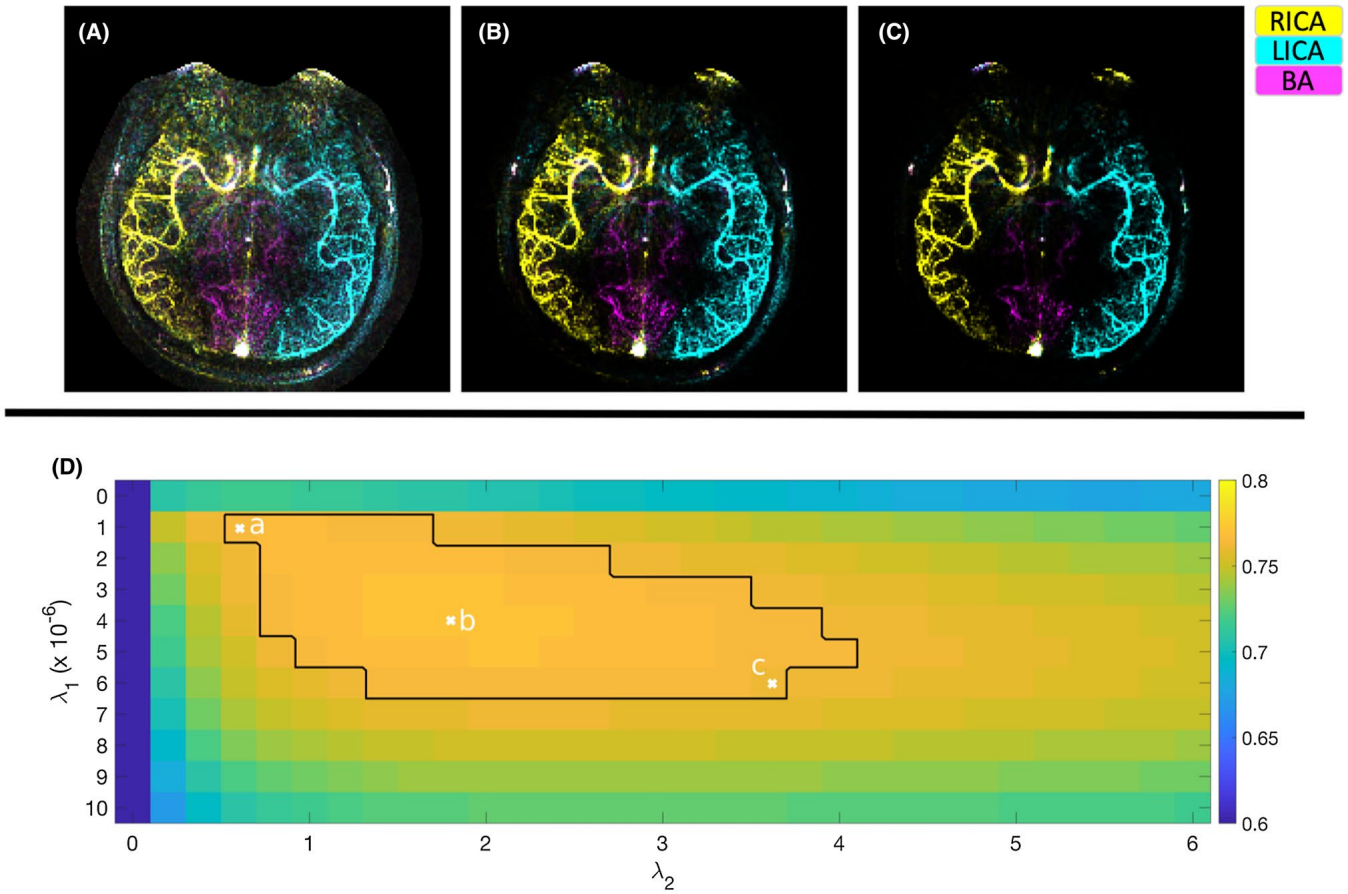
The same raw data set (1 of the in vivo vessel‐encoded R = 34 subsets) reconstructed with 3 different combinations of regularization factors: minimal regularization, and therefore more noise (A); optimal regularization based on the average correlation coefficient (B); and maximal regularization, resulting in a heavily denoised reconstruction (C). (D) shows the average correlation coefficient across all subjects at each combination of regularization factors. The black border in (D) represents the area where the reconstruction was within 1% of optimum, and the “x”s denote the regularization factors used for (A), (B), and (C)

The effect of varying the regularization factor within the overall optimal range was varied sensitivity and specificity (i.e., reduced noise against the cost of losing visibility of small vessels). An example of this tradeoff is shown in Figure 5. For the sake of comparing VE with non‐VE reconstructions in an unbiased way, the group optimum regularization factors for each acquisition method were used for all further analyses. The optimal regularization factors for each acceleration factor and subject or noise level are summarized in Supporting Information Table S1.

**Figure 6:**
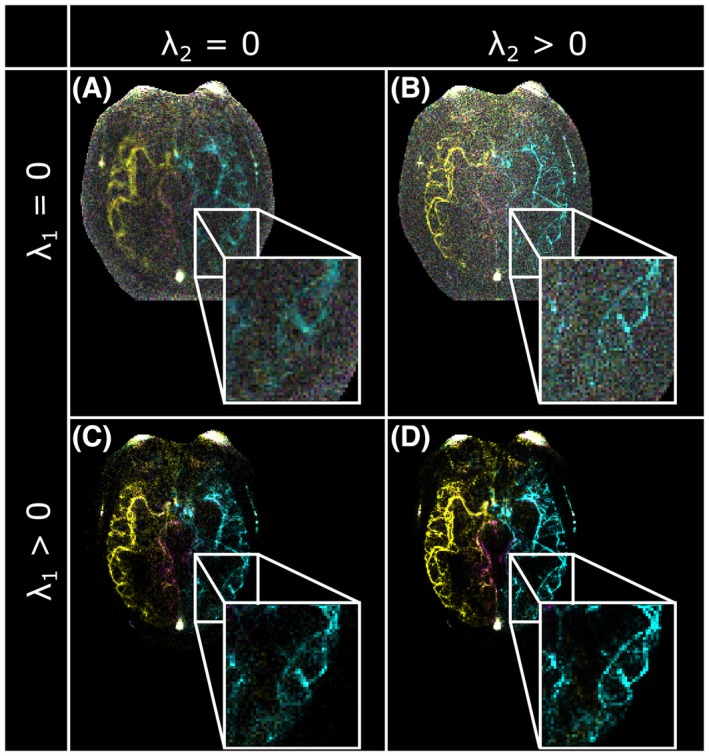
An example of the R = 34 VE‐ASL reconstruction (temporal average) with no regularization (A), only the L_2_ temporal smoothness constraint (B), only the L_1_ sparsity constraint (C), and both regularizing terms included (D). The L_2_ term sharpens the vessels, and the L_1_ term denoises the background. Both regularizing terms improve the overall reconstruction quality

Both regularization terms improved the overall reconstruction quality in all cases. Average correlation coefficients for the in vivo data were improved 4.6%‐55.2% by having a nonzero *λ*_2_, 2.3%‐8.9% by having a nonzero *λ*_1_, and 15.3%‐98.7% by having both regularization factors be nonzero. Similarly, in simulations with nonzero noise, a 1.0%‐13.0% improvement was observed for nonzero *λ*_2_, 0.1%‐10.0% improvement for nonzero *λ*_1_, and 2.2%‐88.3% improvement by having both regularization factors be nonzero. Visually, the value of having both regularizing terms is shown in a sample reconstruction in Figure 6, with nonzero *λ*_2_ causing better delineation of the vessels, and nonzero *λ*_1_ reducing noise and noise‐like artifacts.

### 3.4 Comparison of VE and non‐VE at matched scan time

**Figure 7:**
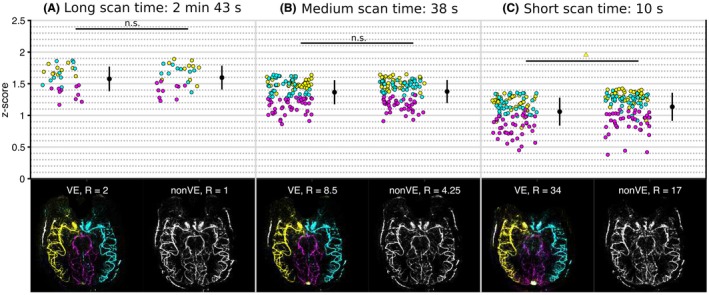
In vivo reconstructions: fully sampled non‐VE versus R = 2 VE (A); moderate acceleration (R = 4.25 non‐VE versus R = 8.5 VE) (B); and high acceleration (R = 17 non‐VE versus R = 34 VE) (C). The bottom row shows a single example of the reconstruction quality for 1 subset in 1 subject for time‐averaged VE and non‐VE. The reconstructions of other subjects can be found in Supporting Information Figures S2‐S6. A version with the inverted grayscale contrast for direct comparison is available in Supporting Information Figure S10

Generally, no statistically significant difference in image quality was found between VE and scan time–matched non‐VE images in vivo despite VE requiring a factor of 2 higher undersampling. A single exception was the high‐acceleration RICA, where the non‐VE correlation coefficients were marginally higher (*P* < .01). The in vivo results are displayed in Figure 7.

Qualitatively, the VE images also looked comparable with the non‐VE images acquired at the same acquisition time (example shown in Figure 7; reconstructions of the other subjects are available in Supporting Information Figures S5‐S9). At high R, a loss of faint features compared with the ground truth was apparent for both non‐VE and VE, and some subsets included artifacts potentially caused by motion. Animations of the dynamic blood flow are available in the Supporting Information Videos [Link], [Link], [Link], [Link], [Link]. Supporting Information Videos [Link], [Link], [Link], [Link], [Link] show the same data but with applied inflow subtraction,^24^ to show inflow instead of outflow of the bolus for better visualization .

**Figure 8:**
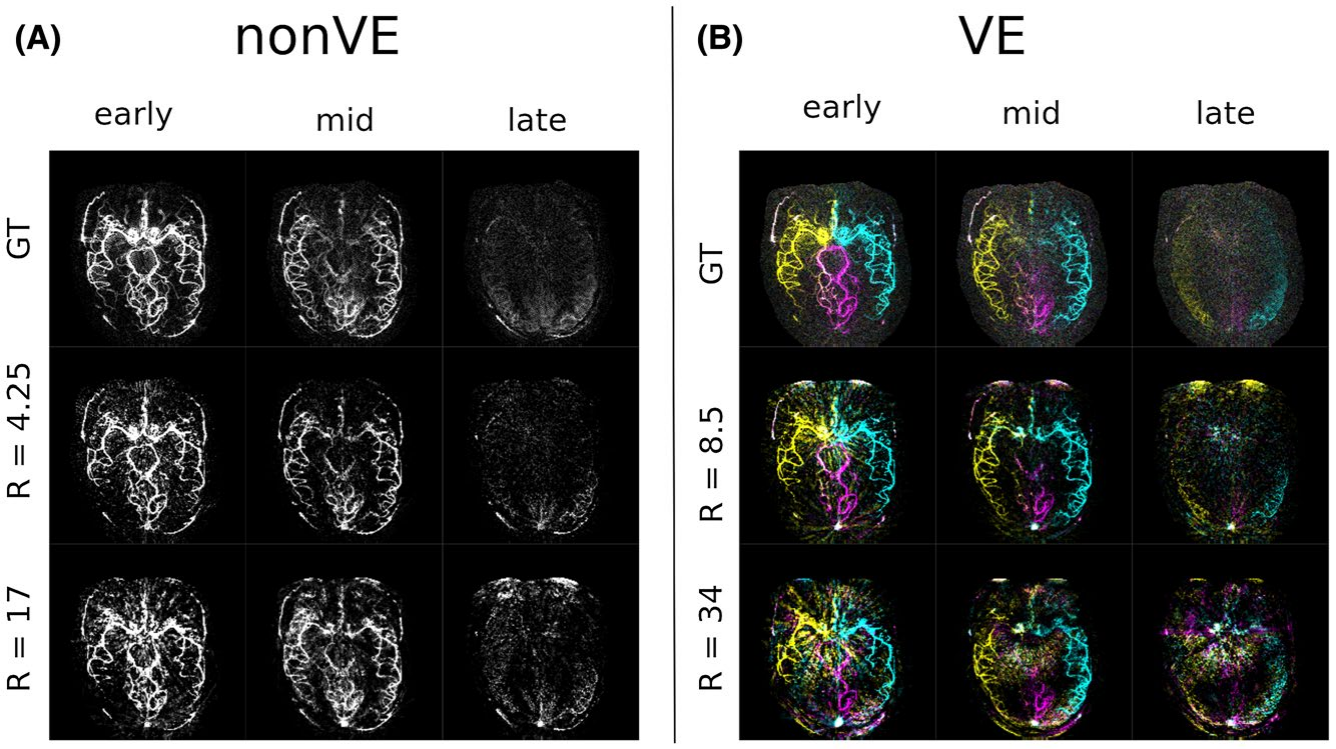
Temporal dynamics in an example subject at varying acceleration factors for a non‐VE acquisition (A) and a VE acquisition (B). The early time point is frame 1, the mid‐timepoint is frame 6, and the late time point is frame 12. A version with the inverted grayscale contrast for direct comparison is available in Supporting Information Figure S11. Animations can be found in the Supporting Information Videos [Link], [Link], [Link], [Link], [Link], and versions with the inflow are visualized in Supporting Information Videos [Link], [Link], [Link], [Link], [Link]

**Figure 9:**
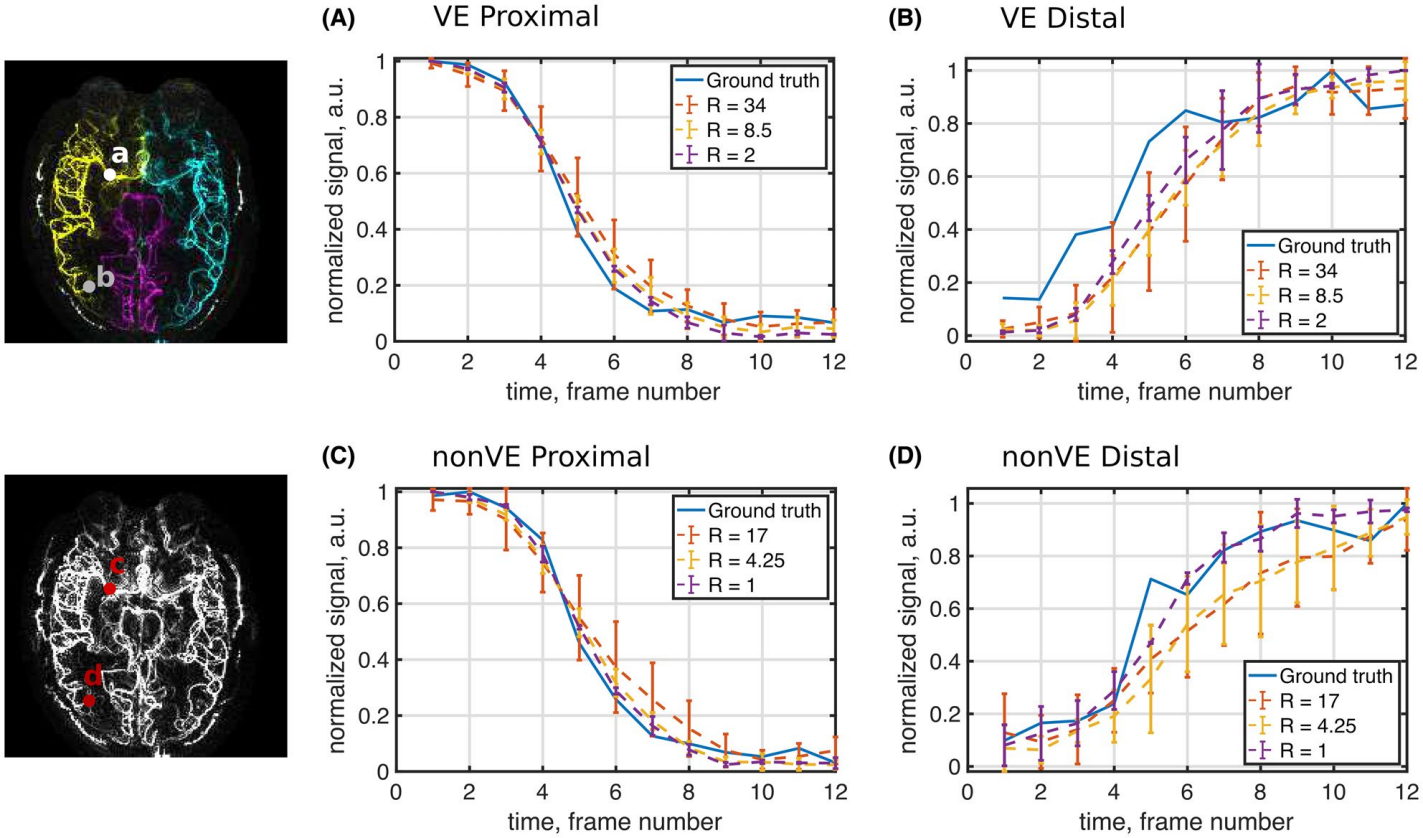
Temporal profile in 2 regions of interest, in a proximal vessel (A,C) and in a distal vessel (B,D), with blood supply from the RICA in 1 subject. The error bars indicate the SD of the signal measured from reconstructions of different subsets of the raw data at each acceleration factor

The temporal dynamics were also well conserved across acceleration factors. The signal was generally well‐preserved in the late frames at moderate acceleration factors, but at the highest acceleration factors some residual aliasing was also present in the later frames (Figure 8) for both the non‐VE and VE images. Figure 9 shows the temporal profile of the signal averaged in two 3 × 3 voxel regions in proximal and distal vessels in a sample subject. In the distal vessel, the SNR is lower and the temporal signal is noisier even in the ground‐truth case. The temporal regularization smooths the signal but preserves overall shape.

### 3.5 High‐resolution images

**Figure 10:**
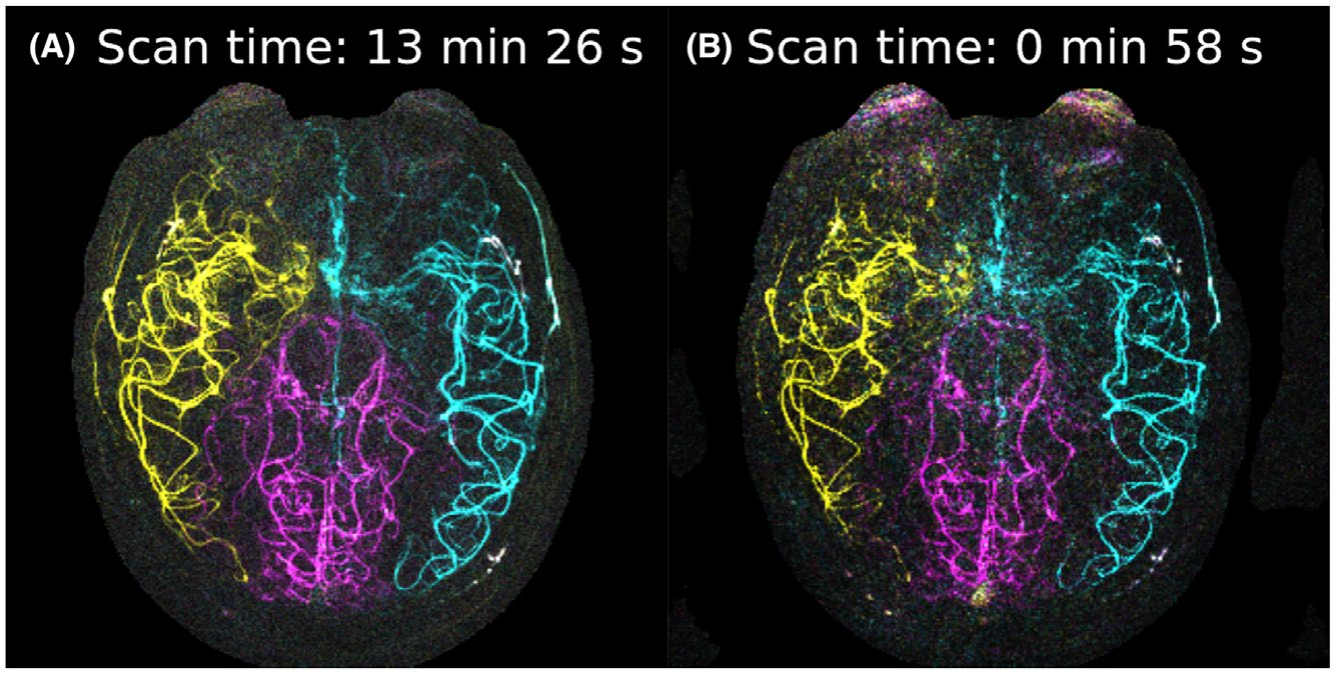
(A) High‐resolution image from oversampled VE scan (R = 0.7). (B) Highly accelerated scan (R = 9.3)

In under a minute (R = 9.3, scan time 58 seconds), very high‐quality images could be acquired at high resolution (Figure 10). The regularization factors used to create this image were on the lower end of the 1% optimal region (*λ*_1_ = 0.000002, *λ*_2_ = 2.0), as higher regularization factors removed many of the fainter vessels to prioritize noise removal.

## 4 DISCUSSION AND CONCLUSIONS

In this study we have shown that the additional sparsity provided by VE allows us to generate vessel‐selective dynamic angiograms in the same time required for conventional ASL angiography without a loss of image quality. The proposed reconstruction method does not rely on a specific type of labeling and could be used with pulsed ASL preparations as well as the pseudo‐continuous ASL approach studied here.

### 4.1 Role of relative sparsity in compressed sensing and VE‐ASL

As hypothesized, the simple simulation experiment showed that relative sparsity (proportion of nonzero voxels to total number of voxels reconstructed) can drive a L_1_‐regularized reconstruction. This agrees with the underlying theory of compressed sensing.^14^ How much relative sparsity drives the reconstruction quality compared with other factors such as SNR, and the spatial distribution of nonzero voxels, are topics worth further consideration. The theoretical and practical limitations of this method in gaining extra information “for free” in a system in which relative sparsity increases should be explored further, beyond this initial feasibility study.

One potential extension is applying this method to VE‐ASL with labeling above the circle of Willis, which would require more encodings because there are more vessel branches, but each decoded image would be sparser, allowing for potentially higher acceleration. However, practical issues, such as achieving an ideal Hadamard encoding for more complicated vessel geometries, might limit the improvements that the increased sparsity alone buys. A strategy for optimizing encodings for complex geometries has, however, been proposed previously.^25^

To further aid reconstruction, the nonsparse nature of the static tissue needs to be considered. Although good images of the static tissue signal are not necessary from a clinical perspective, the static tissue component needs to be reconstructed well, to correctly decode the blood signal. In this work, the static tissue signal was treated just like the blood signals in reconstruction. However, further improvements could be achieved by applying a sparsifying transform, such as the wavelet transform or spatial total variation constraints, as it is not intrinsically sparse in image space like the blood vessels. In similar work on non‐VE pulsed ASL angiography, wavelet regularization on the control image was reported to improve vessel delineation.^26^ By transforming the static tissue to a sparser domain, it should allow for better reconstruction in a compressed‐sensing framework.

### 4.2 Regularization in the temporal domain

In the same vein as increasing the spatial dimensionality by increasing the number of vessels encoded, the dynamic acquisition also allows us to take advantage of structure and redundancy in the temporal domain. In this work, the L_2_ norm of temporal finite differences was used to regularize the reconstruction. It greatly improved the reconstruction results beyond the L_1_ spatial sparsity alone. We observed that the introduction of the temporal regularization in particular improved spatial delineation, which could be explained by the sharing of k‐space information across time frames. In previous studies, temporal constraints have been enforced by using sliding‐window acquisitions^27^ or with compressed sensing using L_1_ constraints in temporal total variation frameworks,^28^ or in the temporal frequency domain.^29^ These approaches can, however, have unwanted effects such as increased blurring for the sliding‐window method, temporal stair‐casing artifacts for total variation,^30^ or artificially introduced periodic behavior in the temporal frequency domain. Model‐based nonlinear reconstruction is another option that has been explored in perfusion ASL,^31^ and whether any of these approaches could improve reconstruction for VE‐ASL angiography could be studied further.

### 4.3 Tuning the regularization factors

Among the 5 volunteers scanned in vivo, the difference in optimal regularization factors was marginal and there was considerable overlap among their optimal regions. This suggests that once the reconstruction has been optimized for an acceleration factor and imaging protocol, it is robust, although to test this rigorously a cross‐validation approach must be used on a larger number of subjects. Because the optimal regularization factors depend on the properties of the actual data (inherent sparsity and temporal smoothness), we have to be careful with extending this conclusion to patients and other populations. There was some variability in head size and angle of imaging slab, and 2 of the volunteers exhibited considerable mixing of RICA and BA blood due to an asymmetrical circle of Willis configuration, but patients with arteriovenous malformations could have considerably lower image domain sparsity due to the presence of additional abnormal vessels that can affect optimal regularization factors considerably. Further studies in appropriate patient groups are required to determine how generalizable this result is.

In this study, the Pearson's correlation coefficient, *r*, was used to objectively optimize the regularization parameters and define the quality of the reconstruction. We found the Pearson's correlation coefficient to be a robust metric that corresponded well with visual perception of image quality, which was not the case for other metrics that were tested in preliminary work. Normalized RMS error is a straightforward metric but it does not correspond well with perceptual quality, as reported in Akasaka et al.^32^ The structural similarity index^33^ that was developed specifically to correspond with visual perception worked well within a data set to tune the parameters, but was not suitable to compare different data sets, as it was sensitive to the absolute scaling of the signal. Often, the SNR is reported as a summary metric for image quality, but it was also found to be unsuitable for this type of reconstruction, as the nonlinear nature of the reconstruction method can cause high SNR when the image quality is poor, by both attenuating the signal (high bias) and removing all noise (low variance). The masking improved robustness, as otherwise the result was driven primarily by large areas with no signal and regions containing artifacts such as eye motion. Tuning could also be done manually by experts to balance specificity (i.e., noise removal) and sensitivity (preservation of faint signals) for optimal clinical utility.

### 4.4 Feasibility of accelerating VE and non‐VE ASL

These results demonstrate the feasibility of acquiring VE images without increasing the scan time compared with fully sampled non‐VE angiography. Similar image quality was obtained with R = 2 VE and R = 1 non‐VE imaging in vivo. Further reductions in scan time have also been shown to be possible, both for non‐VE and VE‐ASL, with matched image quality. At the highest acceleration factor (R = 17 for non‐VE and 34 for VE), the main features were still visible with scan times as short as 10 seconds, although some loss of faint features and artifacts were observed. The required image quality will depend on the clinical application of the technique. For example, if the scan is acquired to add information about mixing of blood from different sources to other angiographic images, a highly accelerated scan of lower quality might be sufficient, whereas if it were used diagnostically on its own, moderate acceleration factors might be more appropriate (as shown in the high‐resolution data set).

In simulation, the correlation coefficient, *r*, was consistently higher for VE at low and medium R, indicating not only equivalent but also slightly improved performance of VE over non‐VE at matched scan time. However, although statistically significant, this difference was small compared with the effect of increasing or decreasing scan time or varying the SNR. At high R, the performance results varied between being in favor of VE and non‐VE, and differences in the qualitative assessment of the image quality were small. This did not contradict the overall conclusion of this study that VE and non‐VE images of similar quality can be achieved at matched scan times, even at the highest acceleration factor.

Similarly, in vivo, the nonsignificant results at low and medium R indicate similar performance level. One potential reason why non‐VE achieved significantly higher correlation coefficients in the RICA at high R is that 2 of the 5 subjects exhibited mixing of blood supply on the right side. Mixing provides higher SNR for the non‐VE reconstruction, as the signal from multiple origins are added together rather than split into 2 vessel components.

For many clinical applications, it would be desirable to extend this technique to 3D. The main reason why 2D imaging of a single slab was chosen for this feasibility study was to be able to acquire ground‐truth images in reasonable scan times. The extension to 3D is straightforward, using a 3D golden angle radial sampling scheme.^34^ However, to fully sample VE‐ASL in 3D at the same resolution would take well over 8 hours, so no fully sampled ground truth could have been acquired for quantitative assessment. Extending to 3D will increase the relative sparsity further and should therefore allow for higher acceleration.

In conclusion, the lack of any meaningfully image‐quality differences between VE and non‐VE data at matched scan times indicates that vessel‐selective information can be acquired in this way with no cost of scan time or image quality, despite the higher undersampling factors required. High‐resolution VE angiograms can also be reconstructed from less than a minute of scanning data, reducing the scan time by approximately an order of magnitude (R = 9.3).

## CONFLICT OF INTEREST

Dr. Okell is an author of US patent applications relating to a maximum a posteriori Bayesian analysis approach for vessel‐encoded data (exclusively licensed to Siemens Healthcare) and a combined angiography and perfusion using radial imaging and ASL technique, which is similar to the pulse sequence used in this study.

## Supporting information


**FIGURE S1** Image SNR comparison between the in vivo acquisitions and the simulations in a fully sampled nonregularized reconstruction
**FIGURE S2** Subject‐specific masks used in the assessment of image reconstruction. Where blood supply was mixed, the most intense vessel component in the ground‐truth image was chosen
**FIGURE S3** Inverted grayscale version of Figure 3 for more direct comparison of image quality, but with the vessel‐specific information lost
**FIGURE S4** Optimal regularization factors (marked with red “x”) were within the optimal area (within 1% of optimum) for every subject. The color represents how many subjects had optimal reconstruction at each combination of regularization factors
**FIGURE S5** Time‐averaged in vivo reconstruction of subject 1. The top row shows the VE reconstruction with blood originating in the RICA in yellow, LICA in cyan, and BA in magenta, at varying acceleration factors. The bottom row shows the time‐matched non‐VE images
**FIGURE S6** Time‐averaged in vivo reconstruction of subject 2. The top row shows the VE reconstruction with blood originating in the RICA in yellow, LICA in cyan, and BA in magenta, at varying acceleration factors. The bottom row shows the time‐matched non‐VE images
**FIGURE S7** Time‐averaged in vivo reconstruction of subject 3. The top row shows the VE reconstruction with blood originating in the RICA in yellow, LICA in cyan, and BA in magenta, at varying acceleration factors. The bottom row shows the time‐matched non‐VE images
**FIGURE S8** Time‐averaged in vivo reconstruction of subject 4. The top row shows the VE reconstruction with blood originating in the RICA in yellow, LICA in cyan, and BA in magenta, at varying acceleration factors. The bottom row shows the time‐matched non‐VE images
**FIGURE S9** Time‐averaged in vivo reconstruction of subject 5. The top row shows the VE reconstruction with blood originating in the RICA in yellow, LICA in cyan, and BA in magenta, at varying acceleration factors. The bottom row shows the time‐matched non‐VE images
**FIGURE S10** Inverted grayscale version of Figure 7 for more direct comparison of image quality, but with the vessel‐specific information lost
**FIGURE S11** Inverted grayscale version of Figure 8 for more direct comparison of image quality, but with the vessel‐specific information lost
**TABLE S1** Optimal regularization factors for in vivo (A) and simulation (B)

**VIDEO S1** Time course of the dynamic angiograms of subject 1: VE reconstruction in the top row, and scan time–matched non‐VE in the bottom row

**VIDEO S2** Time course of the dynamic angiograms of subject 2: VE reconstruction in the top row, and scan time–matched non‐VE in the bottom row

**VIDEO S3** Time course of the dynamic angiograms of subject 2: VE reconstruction in the top row, and scan time–matched non‐VE in the bottom row

**VIDEO S4** Time course of the dynamic angiograms of subject 4: VE reconstruction in the top row, and scan time–matched non‐VE in the bottom row

**VIDEO S5** Time course of the dynamic angiograms of subject 5: VE reconstruction in the top row, and scan time–matched non‐VE in the bottom row

**VIDEO S6** Inflow subtracted time course of the dynamic angiograms of subject 1: VE reconstruction in the top row, and scan time–matched non‐VE in the bottom row

**VIDEO S7** Inflow subtracted time course of the dynamic angiograms of subject 2: VE reconstruction in the top row, and scan time–matched non‐VE in the bottom row

**VIDEO S8** Inflow subtracted time course of the dynamic angiograms of subject 3: VE reconstruction in the top row, and scan time–matched non‐VE in the bottom row

**VIDEO S9** Inflow subtracted time course of the dynamic angiograms of subject 4: VE reconstruction in the top row, and scan time–matched non‐VE in the bottom row

**VIDEO S10** Inflow subtracted time course of the dynamic angiograms of subject 5: VE reconstruction in the top row, and scan time–matched non‐VE in the bottom row

## Data Availability

The underlying data for all quantitative results can be publicly accessed in the Oxford University Research Archive via the following persistent identifier: http://dx.doi.org/10.5287/bodleian:MP00NdYOY.
