# Supplementary material for: Highly accelerated vessel‐selective arterial spin labeling angiography using sparsity and smoothness constraints
Source: Magn Reson Med. 2019 Sep 19;83(3):892–905. doi: 10.1002/mrm.27979 (PMC6899790; doi:10.1002/mrm.27979)
Supplement: Supplementary file 1 — FIGURE S1 Image SNR comparison between the in vivo acquisitions and the simulations in a fully sampled nonregularized reconstruction FIGURE S2 Subject‐specific masks used in the assessment of image reconstruction. Where blood supply was mixed, the most intense vessel component in the ground‐truth image was chosen FIGURE S3 Inverted grayscale version of Figure 3 for more direct comparison of image quality, but with the vessel‐specific information lost FIGURE S4 Optimal regularization factors (marked with red “x”) were within the optimal area (within 1% of optimum) for every subject. The color represents how many subjects had optimal reconstruction at each combination of regularization factors FIGURE S5 Time‐averaged in vivo reconstruction of subject 1. The top row shows the VE reconstruction with blood originating in the RICA in yellow, LICA in cyan, and BA in magenta, at varying acceleration factors. The bottom row shows the time‐matched non‐VE images FIGURE S6 Time‐averaged in vivo reconstruction of subject 2. The top row shows the VE reconstruction with blood originating in the RICA in yellow, LICA in cyan, and BA in magenta, at varying acceleration factors. The bottom row shows the time‐matched non‐VE images FIGURE S7 Time‐averaged in vivo reconstruction of subject 3. The top row shows the VE reconstruction with blood originating in the RICA in yellow, LICA in cyan, and BA in magenta, at varying acceleration factors. The bottom row shows the time‐matched non‐VE images FIGURE S8 Time‐averaged in vivo reconstruction of subject 4. The top row shows the VE reconstruction with blood originating in the RICA in yellow, LICA in cyan, and BA in magenta, at varying acceleration factors. The bottom row shows the time‐matched non‐VE images FIGURE S9 Time‐averaged in vivo reconstruction of subject 5. The top row shows the VE reconstruction with blood originating in the RICA in yellow, LICA in cyan, and BA in magenta, at varying acceleration factors. The bottom row shows the t [file MRM-83-892-s001.pdf]

Supporting Information Table S1 – Optimal regularisation factors for a) in vivo and b) simulation

**a) IN VIVO**	Subj 1		Subj 2		Subj 3		Subj 4		Subj 5		Overall	
	λ_1	λ_2	λ_1	λ_2	λ_1	λ_2	λ_1	λ_2	λ_1	λ_2	λ_1	λ_2
nonVE R = 1	4.00E-06	4	4.00E-06	4	4.00E-06	2	4.00E-06	2	5.00E-06	2	4.00E-06	2
VE R = 2	7.00E-06	6	7.00E-06	6	7.00E-06	6	7.00E-06	6	7.00E-06	4	7.00E-06	6
nonVE R = 4.25	4.00E-06	4	4.00E-06	4	5.00E-06	4	4.00E-04	3	6.00E-06	3	5.00E-06	3
VE R = 8.5	6.00E-06	5	6.00E-06	4	6.00E-06	4	6.00E-06	5	7.00E-06	4	6.00E-06	4
nonVE R = 17	3.00E-06	1.8	3.00E-06	2.2	4.00E-06	2.2	3.00E-06	1.6	4.00E-06	1.8	3.00E-06	1.8
VE R = 34	1.00E-06	0.8	4.00E-06	2	4.00E-06	2	4.00E-06	2	5.00E-06	2	4.00E-06	1.8

**b) SIMULATION**	$SNR_k = \infty$		SNR_k = 185.7		SNR_k = 92.8	
	λ_1	λ_2	λ_1	λ_2	λ_1	λ_2
nonVE R = 1	0	0	1.00E-06	2	4.00E-06	2
VE R = 2	0	0	1.00E-06	2	7.00E-06	4
nonVE R = 4.25	1.00E-06	0	3.00E-06	1	5.00E-06	3
VE R = 8.5	1.00E-06	0	3.00E-06	2	7.00E-06	3
nonVE R = 17	1.00E-06	0.2	2.00E-06	0.6	4.00E-06	1.4
VE R = 34	1.00E-06	0.2	2.00E-06	0.6	5.00E-06	1.4

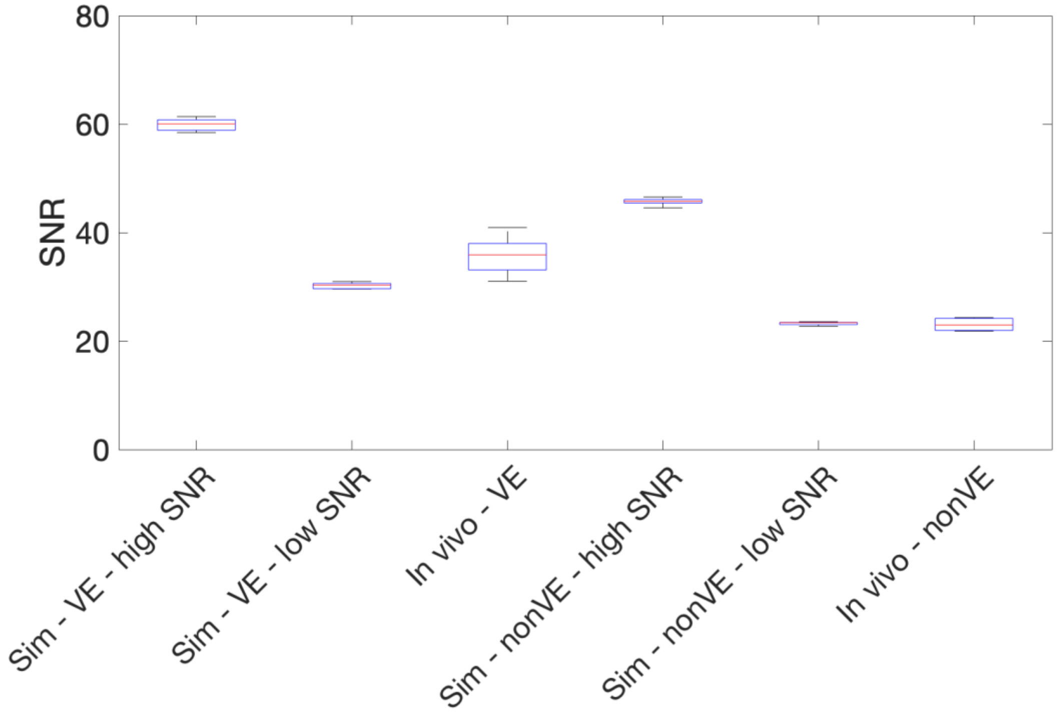

Figure S1 - Image SNR comparison between the in-vivo acquisitions and the simulations in a fully sampled non-regularized reconstruction.

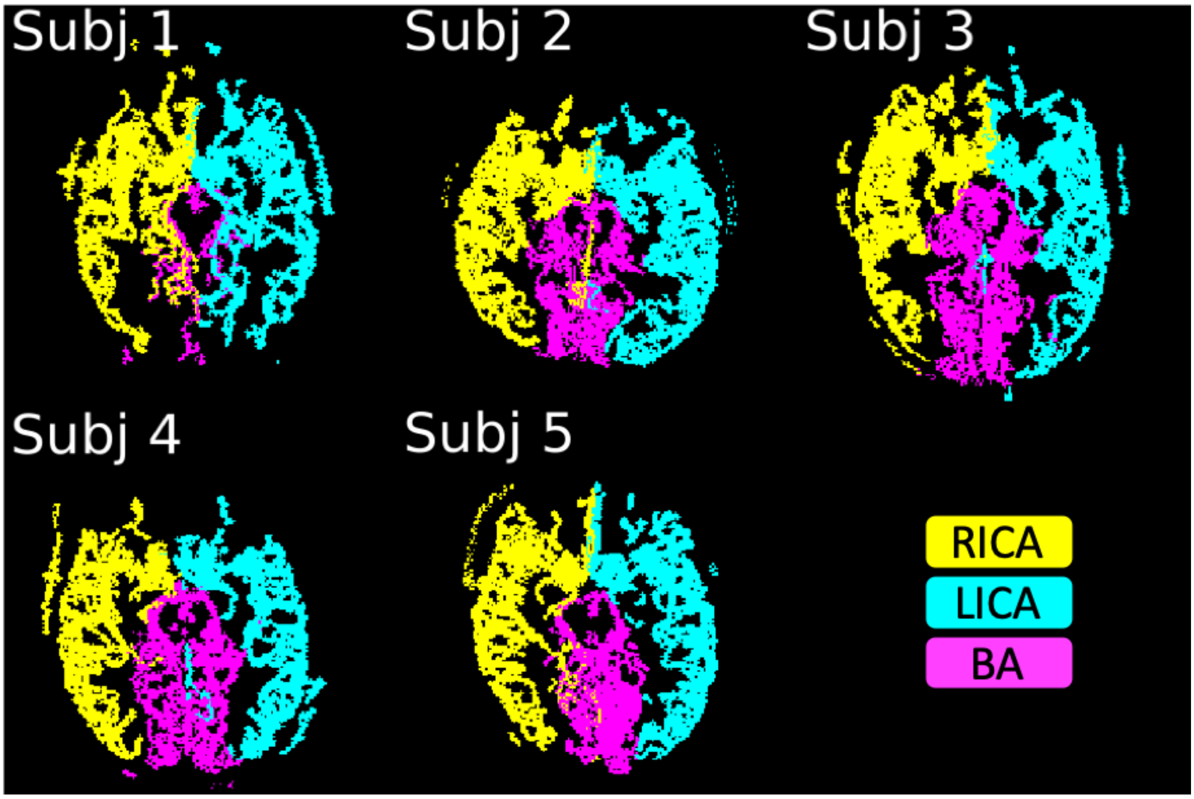

Figure S2 - Subject specific masks used in the assessment of image reconstruction. Where blood supply was mixed the most intense vessel component in the ground truth image was chosen.

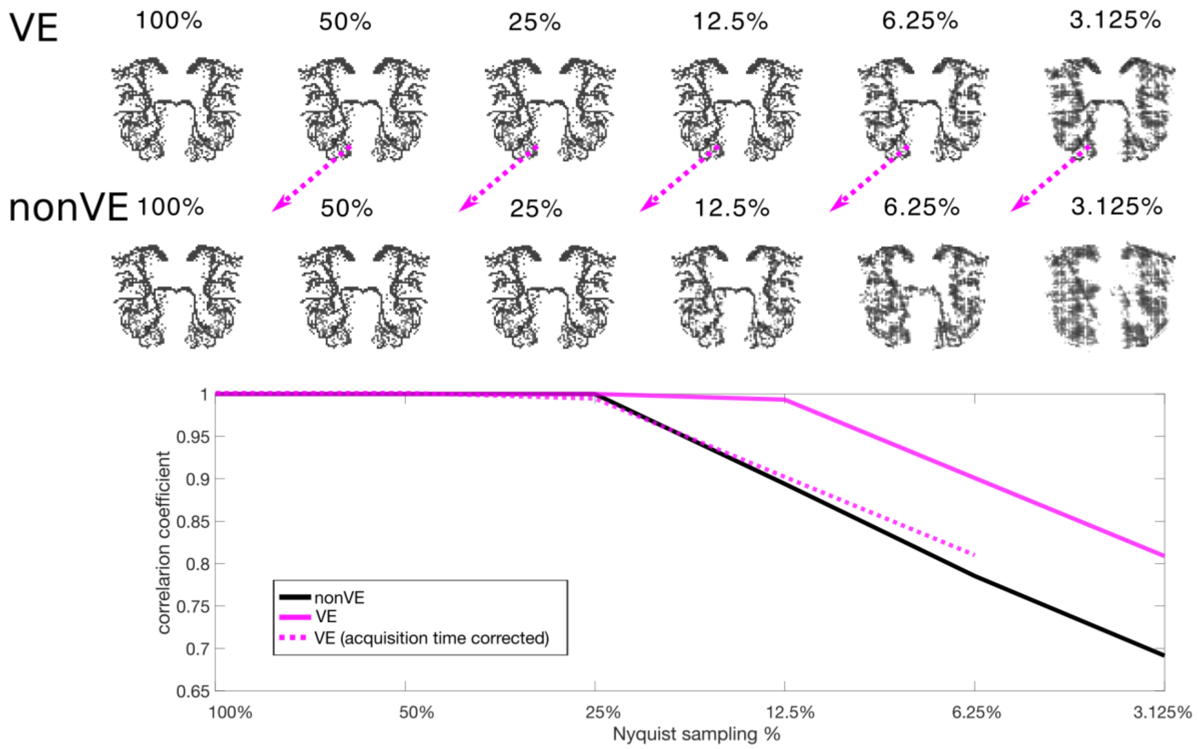

Figure S3 – Inverted grayscale version of Figure 3 for more direct comparison of image quality, but with the vessel specific information lost.

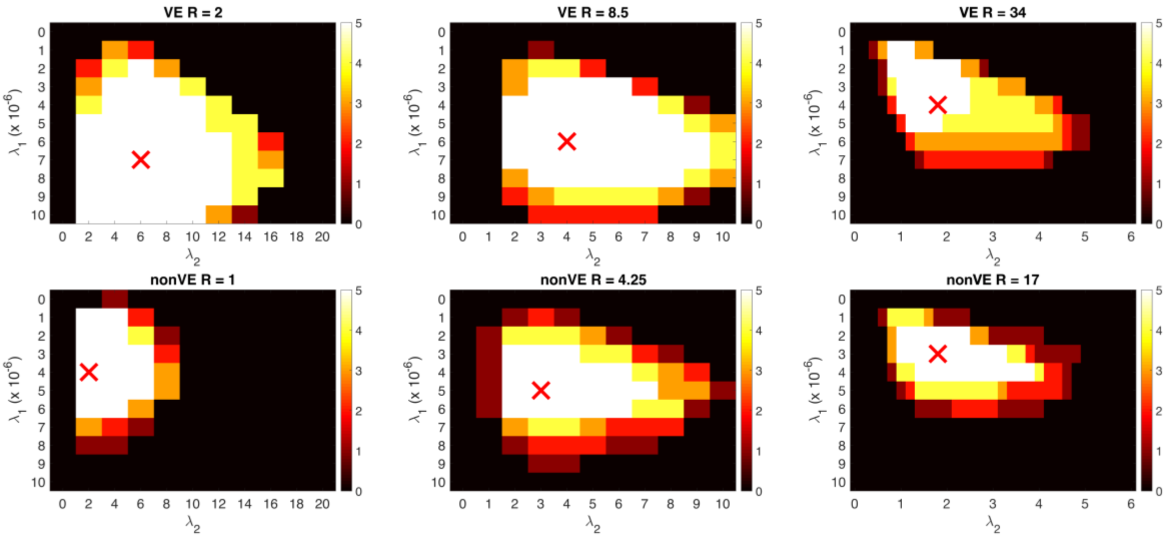

Figure S4 - The optimal regularization factors (marked with red 'x') were within the optimal area (within 1% of optimum) for every subject. The color represents how many subjects had optimal reconstruction at each combination of regularization factors.

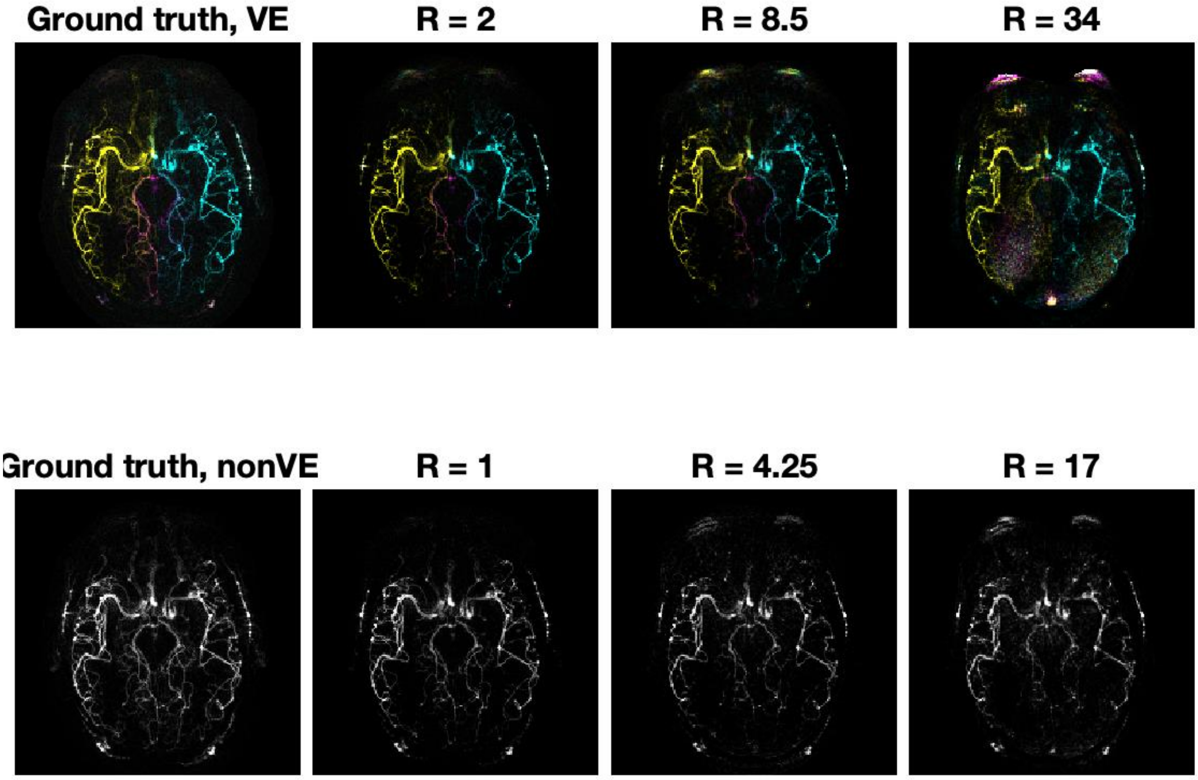

Figure S5 – Time averaged in-vivo reconstruction of Subject 1: The top row shows the VE reconstruction with blood originating in the RICA in yellow, The LICA in cyan, and BA in magenta at varying acceleration factors. The bottom row shows the time matched nonVE images.

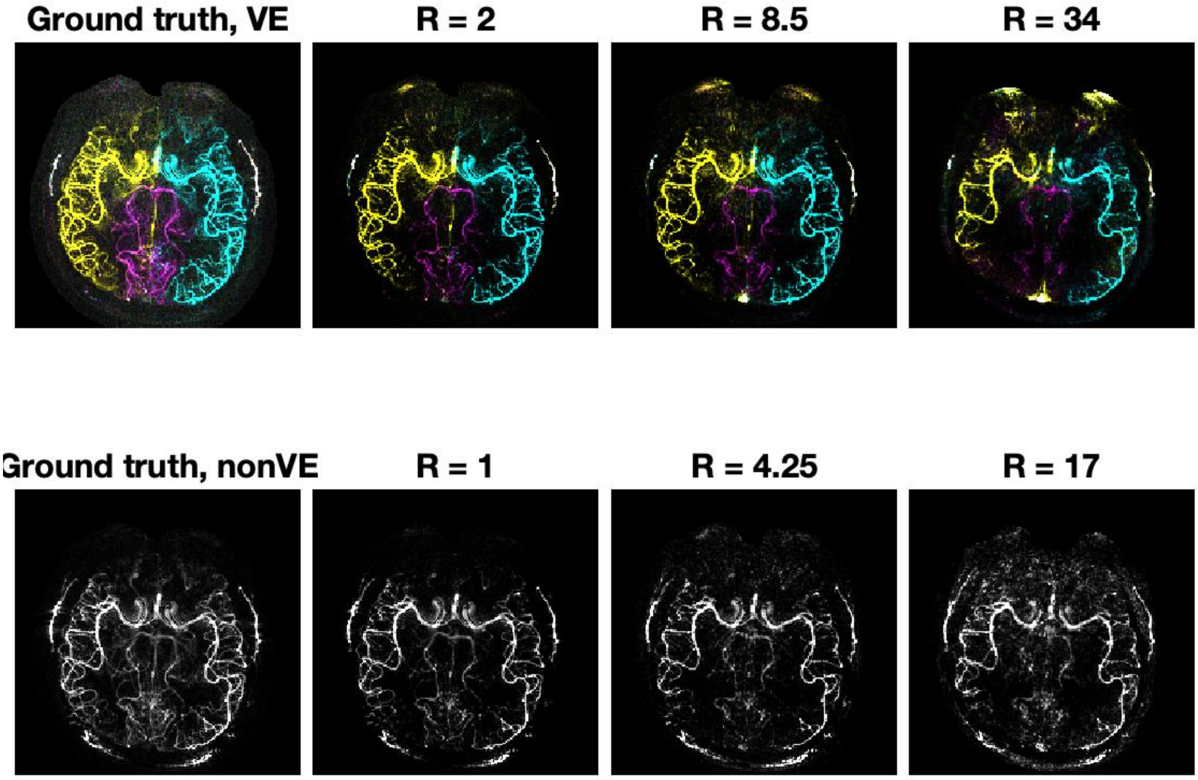

Figure S6 - Time averaged in-vivo reconstruction of Subject 2: The top row shows the VE reconstruction with blood originating in the RICA in yellow, The LICA in cyan, and BA in magenta at varying acceleration factors. The bottom row shows the time matched nonVE images.

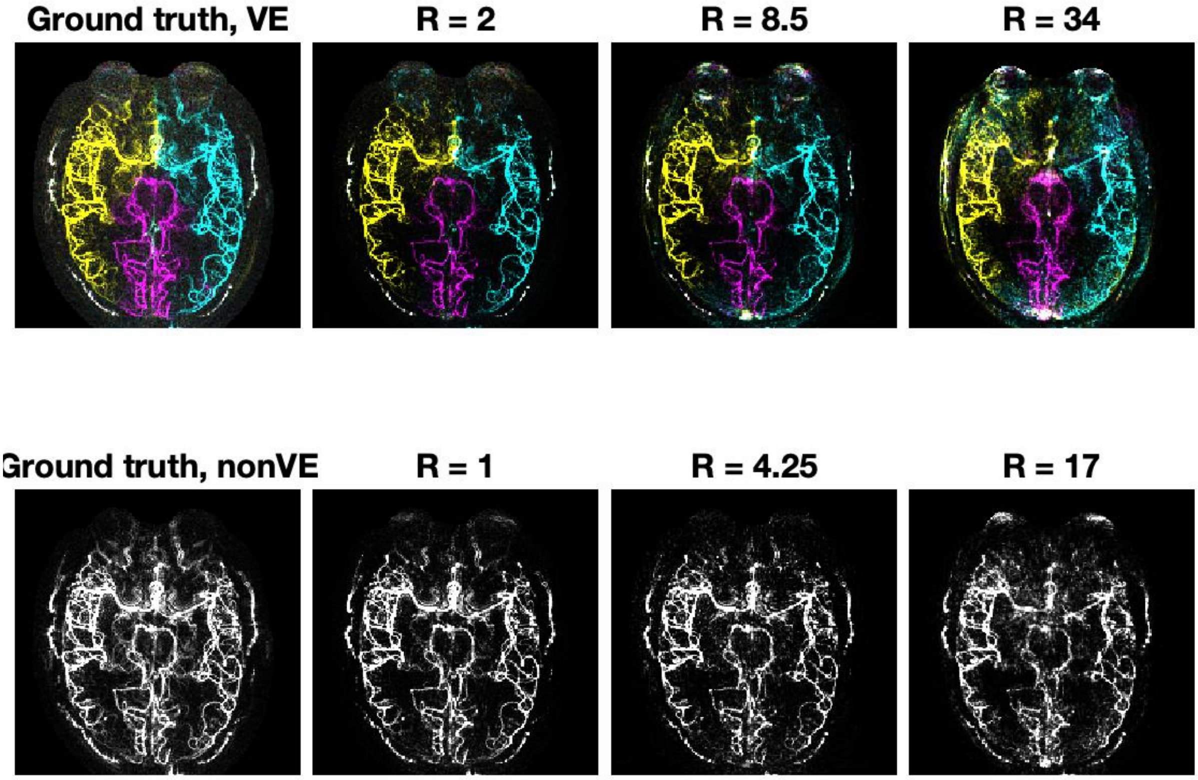

Figure S7 - Time averaged in-vivo reconstruction of Subject 3: The top row shows the VE reconstruction with blood originating in the RICA in yellow, The LICA in cyan, and BA in magenta at varying acceleration factors. The bottom row shows the time matched nonVE images.

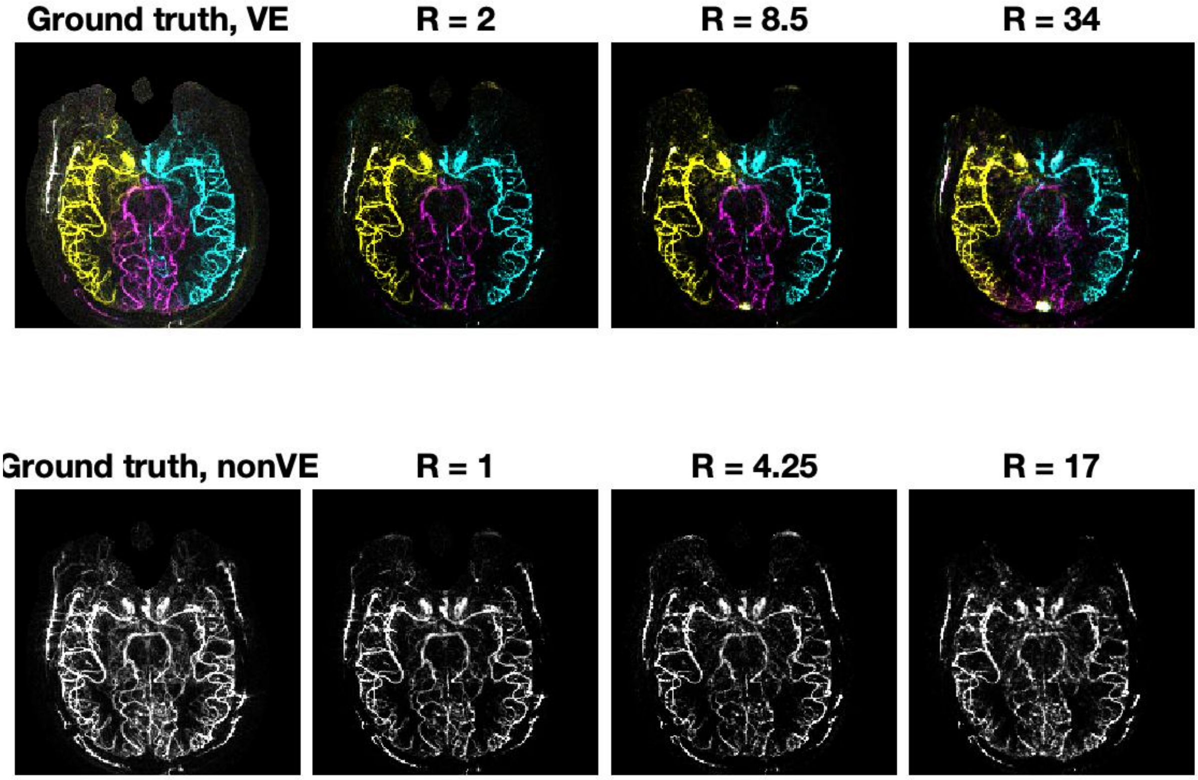

Figure S8 - Time averaged in-vivo reconstruction of Subject 4: The top row shows the VE reconstruction with blood originating in the RICA in yellow, The LICA in cyan, and BA in magenta at varying acceleration factors. The bottom row shows the time matched nonVE images.

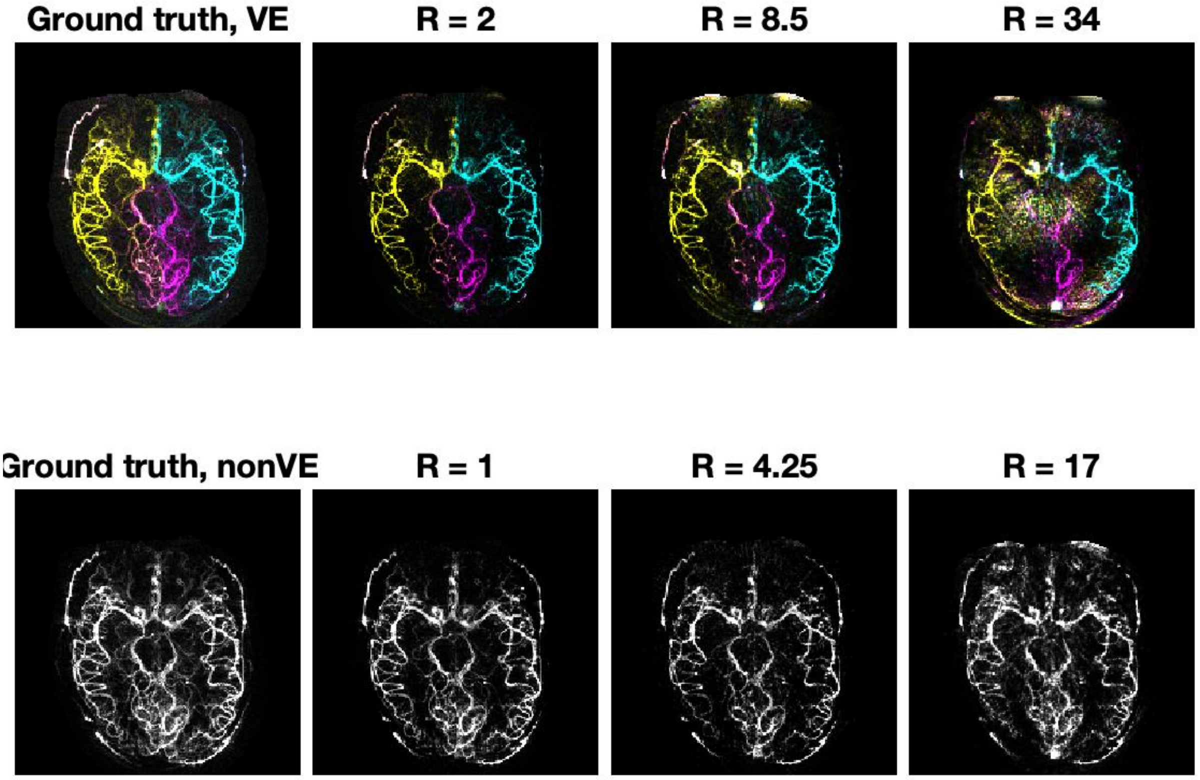

Figure S9 – Time averaged in-vivo reconstruction of Subject 5: The top row shows the VE reconstruction with blood originating in the RICA in yellow, The LICA in cyan, and BA in magenta at varying acceleration factors. The bottom row shows

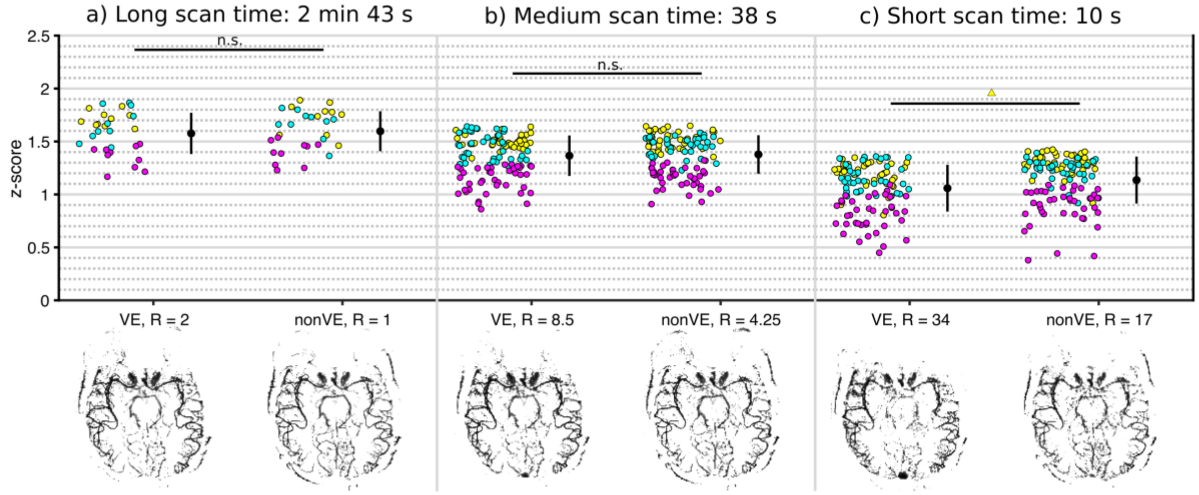

Figure S10 – Inverted grayscale version of Figure 7 for more direct comparison of image quality, but with the vessel specific information lost.

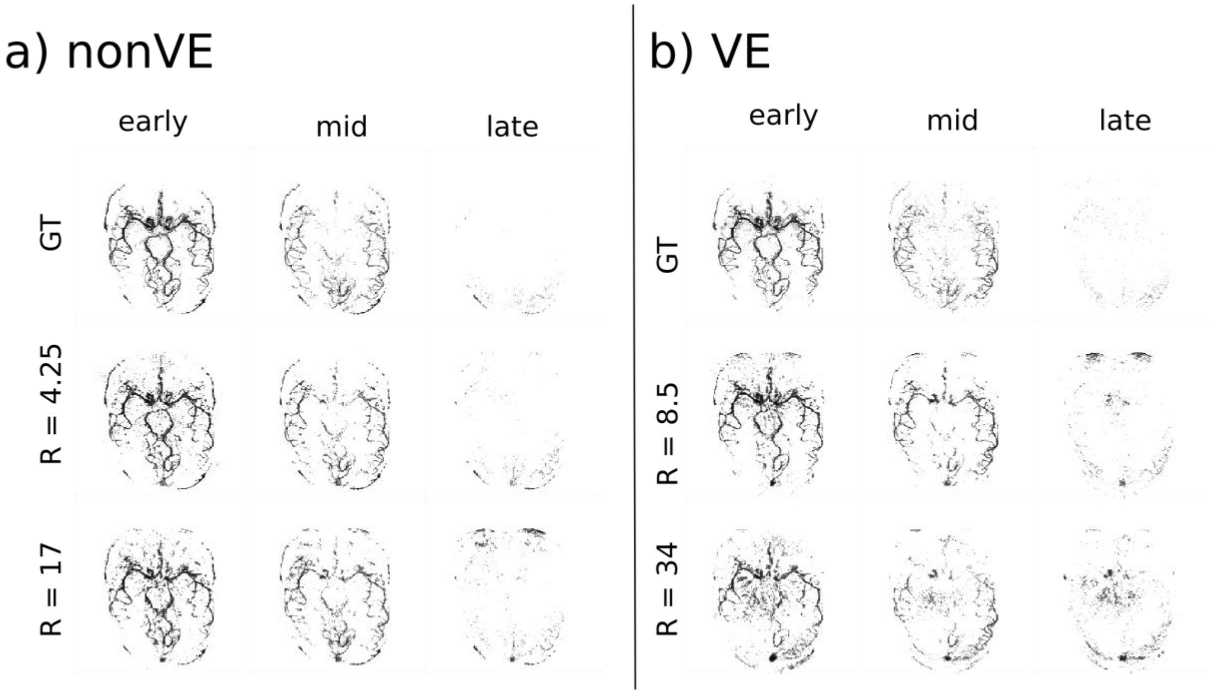

Figure S11 – Inverted grayscale version of Figure 8 for more direct comparison of image quality, but with the vessel specific information lost.
